# Supplementary material for: pomBseen: An automated pipeline for analysis of fission yeast images
Source: PLoS One. 2023 Sep 12;18(9):e0291391. doi: 10.1371/journal.pone.0291391 (PMC10497161; doi:10.1371/journal.pone.0291391)
Supplement: Attachment — Submitted filename: Response to Reviewers.pdf [file pone.0291391.s002.pdf]

Reviewer #1: The authors of “pomBseen: An Automated Pipeline for Analysis of Fission Yeast Images” present a software tool written in MATLAB to perform basic, but important and often time-consuming, steps when analysing fission yeast microscopy images. These steps include cell segmentation and quantification of the nuclear signal (if present). I think the method is well explained and I appreciated the detailed user manual the authors wrote.

However, it is not clear to what extent this software is an improvement of existing methods. I think it is still a valuable contribution because it’s always good for the end user to have multiple options.

To make it clearer to the reader on it can contribute to the image analysis, the authors should address three main points:

1. A proof-of-concept biological experiment is missing. The authors should try to replicate some existing measurements to show that indeed pomBseen can be used to obtain biological insights. A simple experiment that I can think of is to show nuclear size scaling with cell volume.

> The requested analysis is now included in Figure 2G.

Also, why is it important to compute the fluorescence intensity of the nuclear signal? Does it correlate with some biological information?

> One purpose of pomBseen is to measure fluorescence intensity as a proxy for fluorescently-tagged protein concentration. Thus, we report both nuclear and total cellular fluorescence from all fluorescent channels.

2. A benchmark of the segmentation performance against state-of-the-art algorithms. This benchmark should include a comparison to the latest convolutional neural networks available. I can suggest YeaZ and Cellpose for the bright-field, and StarDist and Cellpose for the fluorescence signal. For a benchmark example see the YIT benchmark and MOTA metrics http://yeast-image-toolkit.biosim.eu/pmwiki.php.

> The proposed analysis is not relevant to our work. Our approach is benchmarked against manual annotation and preforms well. We make no claims about performance relative to other computational approaches.

3. To make it more accessible to the reader, the authors should expand the text with more information. Specifically, I propose the following changes:

a. Abstract should start with a couple of sentences to describe the application field, why fission yeast is a good model organisms and what kind of problem there is that pomBseen is solving

> Done.

b. Introduction should start with a description of the biological importance of fission yeast as a model organism and why it is important to study the nucleus using fluorescence microscopy (and what are the advantages of this technique)

> Done.

c. In the introduction, the authors cite some of the existing software, but some other important ones are missing.

The additional software references have been added, but they are not listed by name. This manuscript as not meant as review of currently available segmentation approaches.

Additionally, don't just add the references, but write the name of the software. It makes it easy for the reader to know what you are writing about, without searching in the references. Please cite the following papers:
• StarDist (DOI: https://doi.org/10.1007/978-3-030-00934-2_30)
• Cellpose (DOI: https://doi.org/10.1038/s41592-020-01018-x)
• Cell-ACDC (DOI: https://doi.org/10.1186/s12915-022-01372-6)
• The algorithms used in the YIT benchmark (http://yeast-image-toolkit.biosim.eu/pmwiki.php)

Minor comments:

- Writing "brightfield phase-contrast image" is wrong. Either it is phase-contrast OR brightfield. Maybe the authors wanted to write "wide-field" instead of "bright-field"? On the other hand, the images look bright field to me. Please explain.

The images are bright-field and are now described as such.

- In Figure 1, it is not clear how the image after thresholding (step 3.) can be processed to obtain image shown in step 4. Please add that there is a fill holes step

Done.

- In Figure 1 after ClearBorder step, why are some cells touching the bottom and right border still there? Is this a crop of the top-left part of a larger image? Please specify.

Yes, the displayed images are cropped from the top-left of the full images. This manipulation is now explained.

- In Figure 2 it is not clear how the intensities are calculated in Fiji. Are there masks generated by pomBseen used in Fiji or are the cells segmented manually? Are those mean intensities? Please specify.

The cells were manually segmented and mean intensities are reported, as is now stated in the figure legend.

- Please separate Figure 2 in panel A and B.

Done.

Reviewer #2: This is fine study carried out by authors. However, few comments can further improve the quality of this work.
1. The abstract of article need to be refine. (1) Clearly mention the problem faced in this filed (2) What is your contribution and (3) how this method reliable for fission yeast micrographs than standard other methods available?

Done.

2. The dataset used for this experiment must be referenced and explain how many images part of your experiment?

Done.

3. Convexity filter to remove miss-segmented cells explanation needed more how it help to increase your segmentation results.

> The description of how the convexity filter is applied had been modified in section 6 of the methods and the line separating properly segmented cells from improperly segmented cells and background regions has been labeled in Figure 3B.

4. The figure 2 and 3 have multiple figures. Split each figures into figure 2(a) and figure 2(b) etc. similar for figure 3. Moreover, explain each figure in text separately.

> Done.

Reviewer #3: The authors propose “pomBseen” as a new image analysis pipeline for fission yeast micrographs capable of identifying cells and extracting cell sizes and fluorescent-intensity parameters. The method takes in user-selected images and metadata as inputs, and it produces a CSV file output containing results that are indexed by cell and ready for analysis. The method is implemented in MATLAB and combines functions from existing image processing libraries into a user-friendly program. The method’s automatic segmentation and measurement methods are highly correlated with manual measurements, and the user is provided opportunity to supervise the method discard problematic cells.

In my assessment, pomBseen offers a substantial improvement over existing image analysis pipelines for fission yeast micrographs in several ways. The method achieves close correspondence with human measurements, and the implementation is accessible and flexible. That said, there are a few ways in which the manuscript could be improved prior to publication:

Major issues:

- The underlying data and examples that are used to validate the proposed method are not clearly described in the manuscript. You display results for two different image of manually segmented cells (Fig 2 and Fig 3), but it is unclear whether these images were specially chosen for illustrative purposes, or whether they are representative of the method’s performance more broadly. I ask that you include a more detailed and systematic assessment of the method’s performance over a range of images that are representative of scenarios where others might use the method.

> We now include three additional images representing a range of cell sizes and describe how the data was acquired and state that they are representative of data for which we designed pomBseen.

- The observed correlations between length measurements from pomBseen and human measurement are compelling, but these results could be better assessed in the context of other object identification applications if the authors were to report the intersection over union (IOU) of the automatically segmented cells compared to the manually.

> We did not segment the images manually. We only measured their lengths manually. Therefore, we are unable to report an IOU comparison.

- From the example and information given, it’s unclear what the false positive and true positive rates of the proposed method are. You briefly describe filtering techniques intended to remove objects whose make them unlikely to be pombe cells, but it isn’t clear at what rate other false positives might occur. Even though users having the opportunity to manually remove erroneous

results, it seems that the efficacy of the proposed method to obtain a sample that is equivalent to manual segmentation depends upon the rate at false positives occur and need to be manually excluded.

> The rate of false positives is very low across a wide variety of images. The rate of false negatives is very sensitive to image quality and can range from as little as 5%, as in Figure 2C-E, to almost 100% in poor-quality images. These considerations are now discussed in the manuscript.

Minor issues:

- It's common for machine learning approaches, such as this one, to work best for the data and conditions they were developed under. In this regard, you mention "Although pomBseen was written specifically for DeltaVision fluorescence microscopes and uses its manufacturer-specific data formats, pomBseen should be compatible with a wide range of open access and proprietary image formats, some of which will also include calibration data for the images". I'm curious if you've investigated this claim by applying the proposed method to images obtained by other devices or in different formats. If so, it would be worthwhile to provide an indication of how performance compares.

> No, we have not.

- Similar to the previous point, in Step 6 of the methods you describe an equation for filtering out anomalous cells using aspect ratios. Were the parameters of this equation estimated using your data? Additional information would be helpful if others wanted to adjust these defaults to better calibrate pomBseen (as needed) for their own applications.

> Yes, the parameters were generated from our data, although not the data analyzed in this paper. As now described more explicitly in Section 6 of the Methods, the parameters can be adjusted in the Parameter.m file.

- The caption for Figure 3 is brief and the methods section where it is referenced doesn't fully describe the two components of the figure.

> Both have been expanded.

- There are several paragraphs in the results and discussion describing components of the output CSV file. I felt like these seemed out of place, and that information could be better presented in a table, figure, or example.

> An example of the CSV output is now included as Table S1 and is referenced in the text.
