# Supplementary material for: pomBseen: An automated pipeline for analysis of fission yeast images
Source: PLoS One. 2023 Sep 12;18(9):e0291391. doi: 10.1371/journal.pone.0291391 (PMC10497161; doi:10.1371/journal.pone.0291391)
Supplement: Attachment — Submitted filename: Response to Reviewers v3.pdf [file pone.0291391.s003.pdf]

Reviewer #1: I have nothing to add. Benchmarking against other methods cannot be skipped. It's quite simple to do and it takes max one day. Run YeaZ or cellpose, run your model (which does segmentation since it's clearly shown in Figure 1), compute IoU and compare.

As explained in our previous submission, we acknowledge that such a comparison would be a useful exercise, however that is not the goal of our work. Our purpose in creating and publishing pomBseen is to provide a tool for the analysis of our image data that is as good as our current standard of analysis, which is manual measurement using ImageJ. I am confident that we have done so and the other two reviewers agree. Were we submitting to a journal specializing in automated image analysis, the reviewers concerns might be more germane, but one of the defining characteristics of PLoS ONE is that it is a forum in which one can publish their work, as long as it is original and rigorous, as they conceive of it, not as others think it should be done.

Reviewer #2: The author(s) of article taken all my comments into careful consideration and have made improvements to the manuscript accordingly. The revised article improved the overall quality of the manuscript.

We appreciate the reviewers acknowledgement of the our revision.

Reviewer #3: It is my opinion that the work as it currently stands is suitable for publication. While I agree with the other reviewer that benchmarking would be a worthwhile addition, I also believe that the authors have provided enough for future researchers to perform that work if they seek to compare the efficacy of different tools/methods.

We appreciate the reviewers acknowledgement of the our revision, and emphasize their contention that our manuscript is appropriate for publication in PLoS ONE.
