# Supplementary material for: pomBseen: An automated pipeline for analysis of fission yeast images
Source: PLoS One. 2023 Sep 12;18(9):e0291391. doi: 10.1371/journal.pone.0291391 (PMC10497161; doi:10.1371/journal.pone.0291391)
Supplement: Attachment — Submitted filename: Review of Ohira and Rhind.pdf [file pone.0291391.s005.pdf]

Review of Ohira and Rhind Revision 4

This manuscript provides a versatile, efficient and accurate program to automate measurements of micrograph images of fission yeast. I understand some reviewers feel the output should be compared to other software, but I do not agree. The authors have demonstrated in Fig 2 that the output is highly accurate regarding cell length and nuclear intensity. The community of biologists that study fission yeast will find this program to be extremely useful.

Specific comments

1. No page number or line numbers are provided. This makes it hard to describe comments. I will consider the abstract and Intro to be page 1.
2. Page 1, 9 lines from bottom. Add space to correct Tables 1and S1.
3. Page 3, text- pomBseen should be compatible with a wide range of open access…" I would like to see some more specificity here to help the reader understand which microscope systems are compatible. Can the authors provide more details.
