# Supplementary material for: pomBseen: An automated pipeline for analysis of fission yeast images
Source: PLoS One. 2023 Sep 12;18(9):e0291391. doi: 10.1371/journal.pone.0291391 (PMC10497161; doi:10.1371/journal.pone.0291391)
Supplement: Attachment — Submitted filename: Response to Reviewers v4.pdf [file pone.0291391.s007.pdf]

Reviewer #4:

In this manuscript, the authors present an image analysis tool for microscopic images of fission yeast, allowing for automated cell segmentation and quantification of fluorescent signals. As the other reviewer suggested, a comparison with other existing methods would strengthen the manuscript. However, there is no standard method, and many fission yeast labs still perform measurements manually.
I have only a few minor comments.

It would be helpful to provide examples of analysis of images taken with systems other than deltavision to demonstrate the versatility of pomBseen.

> We do not have access to another comparable microscope system. However, we have confirmed that pomBseen works with TIFF files. Therefore, pomBseen should work with all systems that can save images in TIFF format. We have now explicitly stated the general applicability of pomBseen in the manuscript.

What are DV files? Is it a specific file type for the deltavision microscope?

> DV files are the proprietary image file type produced by DeltaVision microscopes.

Reviewer #5:

I am a new reviewer and has seen the previous reviews and editor comments. In my opinion this paper can be published in PLoS One without a benchmarking and it provides a usedul and valid tool (as validated by comparison with manually analysed data). However, I suggest this sentence in the introduction to be better qualified, in what way the other tools were not robust or simple? Is there a particular experimental set up this tool would be more suitable. Please be as specific as you can be about the tools tried.

"However, we found that none of the packages available when we started this project provided a simple, robust solution for the quantitation of our fluorescent images."

> I would rather not call out in print the specific packages that we tried, because I cannot be confident that their failures in our hands were not our fault. However, we are now more specific with the following language, which describes our general problems and makes it clear that we are not referring to any of the packages published after 2019.
>
> "However, we found that none of the packages available in 2019, when we started this project, provided a simple, robust solution for the quantitation of our fluorescent images. In particular, we found that packages designed for mammalian-cell images performed poorly on fission yeast."
>
> For the reviewers information: We could not get PombeX to run, despite trying multiple hardware configurations and contacting the authors. We found ilastic far too complicated to use, particularly in terms of training. And we found that CellProfiler did a poor job of segmenting our fission yeast images.

Reviewer #6:

This manuscript provides a versatile, efficient and accurate program to automate measurements of micrograph images of fission yeast. I understand some reviewers feel the output should be

compared to other software, but I do not agree. The authors have demonstrated in Fig 2 that the output is highly accurate regarding cell length and nuclear intensity. The community of biologists that study fission yeast will find this program to be extremely useful.

Specific comments
1. No page number or line numbers are provided. This makes it hard to describe comments. I will consider the abstract and Intro to be page 1.

> Page numbers have been added.

2. Page 1, 9 lines from bottom. Add space to correct Tables 1and S1.

> Done.

3. Page 3, text- pomBseen should be compatible with a wide range of open access…" I would like to see some more specificity here to help the reader understand which microscope systems are compatible. Can the authors provide more details.

> Bio-Formats opens over 100 proprietary and open-source image formats, a list of which is available at their website <https://docs.openmicroscopy.org/bio-formats/5.3.4/supported-formats.html>. This information is now explicitly included in the manuscript. I know of no microscope system not included, but we have no objective way of stating that, beyond providing their list of supported formats.

Reviewer #7:

Briefly, the manuscript described development of an automated analysis pipeline, to measure the length and fluorescence intensity of yeast cells and their nuclei. Using programming language MATLAB for image-analysis which is widely spread and is interesting approach, however, there are some issues to be addressed in this current manuscript. My comments are below.

1. The authors should edit figures and results more comprehensive and precise. Figures seems to be insufficient of explanation of the abbreviation without consideration for readers.

> We would be happy to address specific concerns. However, the request to "edit figures and results more comprehensive and precise" is too vague to be useful direction. Moreover, none of the other six reviewers have raised any concerns regarding clarity.

2. The authors should offer a more comprehensive explanation of the improvement points compared to the references presented in the Introduction section. For instance, Baybay EK et al. described the 3D segmentation of radially symmetric cells, which involved the combination of 2D brightfield segmentation with 3D morphological extrapolation using fission yeast (Reference #4). However, the authors did not provide the limitations of this research and a detailed explanation of how their pipeline improves upon the existing approach.

> As we have tried hard to make clear in previous submissions, the purpose of this manuscript, and the work it describes, is not to improve on other automated analysis approaches, but rather to provide a automated approach that is as good as, but much more efficient than, manual measurement. Therefore, we have nothing to say regarding the utility of Pomegranate (Baybay EK et al.) or any other automated analysis pipeline.

3. In the Method section, the authors descripted about the images analyzed for study. A couple of strains of cells from reference (#17), however, the strains of the reference were for functional

examination of the Cdc 13 and Cdc 25 expression. The authors should explain about the reason for choosing the specific strains for their study.

> These strains are frequently used in the lab and serve here as simply convenient examples of the sort of fluorescent images that pomBseen can analyze. There is no specific reason for choosing them.

4. Furthermore, the authors should provide the number of image data used for validating PomBseen and more detailed calculating methods, catering to future users rather than programmers.

> The four images used for validation are shown in Figure 2. It is unclear from the reviewers request what "more detailed calculating methods" they would find useful.

5. The authors should thoroughly describe the significance of employing the MATLAB program for image analysis in comparison to other methods. This aspect constitutes a key strength of the manuscript.

> As explained in the Introduction, MATLAB was chosen because it is a convenient, well-supported programing environment. We did not carefully compare it to other options, so we cannot be more thorough in our comparison.
