# Supplementary material for: pomBseen: An automated pipeline for analysis of fission yeast images
Source: PLoS One. 2023 Sep 12;18(9):e0291391. doi: 10.1371/journal.pone.0291391 (PMC10497161; doi:10.1371/journal.pone.0291391)
Supplement: Attachment — Submitted filename: Appeal Responses.doc [file pone.0291391.s004.doc]

**Response to Initial Reviews**

Reviewer #1: The authors of “pomBseen: An Automated Pipeline for Analysis of Fission Yeast Images” present a software tool written in MATLAB to perform basic, but important and often time-consuming, steps when analysing fission yeast microscopy images. These steps include cell segmentation and quantification of the nuclear signal (if present). I think the method is well explained and I appreciated the detailed user manual the authors wrote.

However, it is not clear to what extent this software is an improvement of existing methods. I think it is still a valuable contribution because it’s always good for the end user to have multiple options.

To make it clearer to the reader on it can contribute to the image analysis, the authors should address three main points:

1. A proof-of-concept biological experiment is missing. The authors should try to replicate some existing measurements to show that indeed pomBseen can be used to obtain biological insights. A simple experiment that I can think of is to show nuclear size scaling with cell volume.

The requested analysis is now included in Figure 2G.

Also, why is it important to compute the fluorescence intensity of the nuclear signal? Does it correlate with some biological information?

One purpose of pomBseen is to measure fluorescence intensity as a proxy for fluorescently-tagged protein concentration. Thus, we report both nuclear and total cellular fluorescence from all fluorescent channels.

2. A benchmark of the segmentation performance against state-of-the-art algorithms. This benchmark should include a comparison to the latest convolutional neural networks available. I can suggest YeaZ and Cellpose for the bright-field, and StarDist and Cellpose for the fluorescence signal. For a benchmark example see the YIT benchmark and MOTA metrics http://yeast-image-toolkit.biosim.eu/pmwiki.php.

The proposed analysis is not relevant to our work. Our approach is benchmarked against manual annotation and preforms well. We make no claims about performance relative to other computational approaches.

3. To make it more accessible to the reader, the authors should expand the text with more information. Specifically, I propose the following changes:

a. Abstract should start with a couple of sentences to describe the application field, why fission yeast is a good model organisms and what kind of problem there is that pomBseen is solving

Done.

b. Introduction should start with a description of the biological importance of fission yeast as a model organism and why it is important to study the nucleus using fluorescence microscopy (and what are the advantages of this technique)

Done.

c. In the introduction, the authors cite some of the existing software, but some other important ones are missing.

The additional software references have been added, but they are not listed by name. This manuscript as not meant as review of currently available segmentation approaches.

Additionally, don’t just add the references, but write the name of the software. It makes it easy for the reader to know what you are writing about, without searching in the references. Please cite the following papers:

• StarDist (DOI: https://doi.org/10.1007/978-3-030-00934-2_30 )

• Cellpose (DOI: https://doi.org/10.1038/s41592-020-01018-x )

• Cell-ACDC (DOI: https://doi.org/10.1186/s12915-022-01372-6 )

• The algorithms used in the YIT benchmark (http://yeast-image-toolkit.biosim.eu/pmwiki.php)

Minor comments:

- Writing “brightfield phase-contrast image” is wrong. Either it is phase-contrast OR brightfield. Maybe the authors wanted to write “wide-field” instead of “bright-field”? On the other hand, the images look bright field to me. Please explain.

The images are bright-field and are now described as such.

- In Figure 1, it is not clear how the image after thresholding (step 3.) can be processed to obtain image shown in step 4. Please add that there is a fill holes step

Done.

- In Figure 1 after ClearBorder step, why are some cells touching the bottom and right border still there? Is this a crop of the top-left part of a larger image? Please specify.

Yes, the displayed images are cropped from the top-left of the full images. This manipulation is now explained.

- In Figure 2 it is not clear how the intensities are calculated in Fiji. Are there masks generated by pomBseen used in Fiji or are the cells segmented manually? Are those mean intensities? Please specify.

The cells were manually segmented and mean intensities are reported, as is now stated in the figure legend.

- Please separate Figure 2 in panel A and B.

Done.

Reviewer #2: This is fine study carried out by authors. However, few comments can further improve the quality of this work.

1. The abstract of article need to be refine. (1) Clearly mention the problem faced in this filed (2) What is your contribution and (3) how this method reliable for fission yeast micrographs than standard other methods available?

Done.

2. The dataset used for this experiment must be referenced and explain how many images part of your experiment?

Done.

3. Convexity filter to remove miss-segmented cells explanation needed more how it help to increase your segmentation results.

The description of how the convexity filter is applied had been modified in section 6 of the methods and the line separating properly segmented cells from improperly segmented cells and background regions has been labeled in Figure 3B.

4. The figure 2 and 3 have multiple figures. Split each figures into figure 2(a) and figure 2(b) etc. similar for figure 3. Moreover, explain each figure in text separately.

Done.

Reviewer #3: The authors propose “pomBseen” as a new image analysis pipeline for fission yeast micrographs capable of identifying cells and extracting cell sizes and fluorescent-intensity parameters. The method takes in user-selected images and metadata as inputs, and it produces a CSV file output containing results that are indexed by cell and ready for analysis. The method is implemented in MATLAB and combines functions from existing image processing libraries into a user-friendly program. The method’s automatic segmentation and measurement methods are highly correlated with manual measurements, and the user is provided opportunity to supervise the method discard problematic cells.

In my assessment, pomBseen offers a substantial improvement over existing image analysis pipelines for fission yeast micrographs in several ways. The method achieves close correspondence with human measurements, and the implementation is accessible and flexible. That said, there are a few ways in which the manuscript could be improved prior to publication:

Major issues:

- The underlying data and examples that are used to validate the proposed method are not clearly described in the manuscript. You display results for two different image of manually segmented cells (Fig 2 and Fig 3), but it is unclear whether these images were specially chosen for illustrative purposes, or whether they are representative of the method’s performance more broadly. I ask that you include a more detailed and systematic assessment of the method’s performance over a range of images that are representative of scenarios where others might use the method.

We now include three additional images representing a range of cell sizes and describe how the data was acquired and state that they are representative of data for which we designed pomBseen.

- The observed correlations between length measurements from pomBseen and human measurement are compelling, but these results could be better assessed in the context of other object identification applications if the authors were to report the intersection over union (IOU) of the automatically segmented cells compared to the manually.

We did not segment the images manually. We only measured their lengths manually. Therefore, we are unable to report an IOU comparison.

- From the example and information given, it’s unclear what the false positive and true positive rates of the proposed method are. You briefly describe filtering techniques intended to remove objects whose make them unlikely to be pombe cells, but it isn’t clear at what rate other false positives might occur. Even though users having the opportunity to manually remove erroneous results, it seems that the efficacy of the proposed method to obtain a sample that is equivalent to manual segmentation depends upon the rate at false positives occur and need to be manually excluded.

The rate of false positives is very low across a wide variety of images. The rate of false negatives is very sensitive to image quality and can range from as little as 5%, as in Figure 2C-E, to almost 100% in poor-quality images. These considerations are now discussed in the manuscript.

Minor issues:

- It’s common for machine learning approaches, such as this one, to work best for the data and conditions they were developed under. In this regard, you mention “Although pomBseen was written specifically for DeltaVision fluorescence microscopes and uses its manufacturer-specific data formats, pomBseen should be compatible with a wide range of open access and proprietary image formats, some of which will also include calibration data for the images”. I’m curious if you’ve investigated this claim by applying the proposed method to images obtained by other devices or in different formats. If so, it would be worthwhile to provide an indication of how performance compares.

No, we have not.

- Similar to the previous point, in Step 6 of the methods you describe an equation for filtering out anomalous cells using aspect ratios. Were the parameters of this equation estimated using your data? Additional information would be helpful if others wanted to adjust these defaults to better calibrate pomBseen (as needed) for their own applications.

Yes, the parameters were generated from our data, although not the data analyzed in this paper. As now described more explicitly in Section 6 of the Methods, the parameters can be adjusted in the Parameter.m file.

- The caption for Figure 3 is brief and the methods section where it is referenced doesn’t fully describe the two components of the figure.

Both have been expanded.

- There are several paragraphs in the results and discussion describing components of the output CSV file. I felt like these seemed out of place, and that information could be better presented in a table, figure, or example.

An example of the CSV output is now included as Table S1 and is referenced in the text.

**Response the Second Round of Reviews**

The major concern of Review 1 is that we have not compared our method to other automated image-analysis methods. I concede that such a comparison would be a useful exercise, however that is not the goal of our work. Our purpose in creating and publishing pomBseen is to provide a tool for the analysis of our image data that is as good as our current standard of analysis, which is manual measurement using ImageJ. I am confident that we have done so.

Direct responses to the reviewer's specific comments are below.

Reviewer #1: I thank the authors for having addressed my comments, I think the manuscript has improved.

However, I don't think they can skip benchmarking their method against other automated tools. And even if comparing to manual segmentation was enough (which is not), the authors only compared the length and not the instance segmentation output of the model.

I have addressed the issue of benchmarking above. I do not understand the point that "the authors only compared the length and not the instance segmentation output of the model". Our approach measures two parameters: cell length and nuclear fluorescence. As, described below, in Figure 2 we compared both: length as manually measured using the ImageJ Line Selection tool and mean nuclear intensity as manually measured using the Freehand Selection tool. So, although we did not manually segment the cells, per se, we did validate the both segmentation steps of pomBseen by comparing them to manual measurements.

This is a standard in the field and without it, the reader cannot properly assess the performance of the model.

Additionally, there are some discrepancies:

1. You mentioned that the "cells were manually segmented", but then you also mentioned that you did not segment the images manually. Even if you did not segment the cells manually, the IoU comparison cannot be skipped.

My statement in my response to the Reviewer that "cells were manually segmented" was careless. In the figure legend, we accurately stated that we "manually quantitated" the cells. As now more fully explained in the figure legend, we manually measured the cell length using the ImageJ Line Selection tool and manually measured the mean intensity of the nuclei using the Freehand Selection tool, neither of which capture the data that would be needed for an IoU analysis.

2. Related to 1, you mentioned that the "Manual ImageJ Intensity" of Figure 2 are mean intensities, but how do you get mean intensities if you did not segment them manually? The length is not enough to get the mean intensities.

We manually measured the mean intensity of the nuclei using the Freehand Selection tool, as now explained in the figure legend.

3. If I am not mistaken, the Figure 2 legend still doesn't say that the intensities are mean intensities. Please add this to the axis label on the figures too.

Done.

4. The reason why you cannot skip benchmarking against other automated methods is that manual analysis can be subjective, as you properly mentioned in the abstract. Therefore, comparing only to manual analysis does not address this manual analysis bias.

We do not aim, nor claim, to be as good as or better than any other automated method. We simply claim to be as good as, but more efficient than, our current method of manual analysis. And I am confident we have robustly demonstrated as much.

**Responses to the Final Round of Reviews and the Editor's Rejection Letter**

Reviewer 1: I have nothing to add. Benchmarking against other methods cannot be skipped. It's quite simple to do and it takes max one day. Run YeaZ or cellpose, run your model (which does segmentation since it's clearly shown in Figure 1), compute IoU and compare.

As explained in our previous responses to Reviewer 1, the proposed benchmarking is not relevant to our work. Our approach is benchmarked against the most robust available ground truth, manual annotation, and preforms well. We make no claims about performance relative to other computational approaches.

To be more explicit, there is no possible outcome from the proposed benchmarking analysis that would contribute to the characterization of pomBseen. We demonstrated that pomBseen robustly recapitulates manual annotation (r^2^= 0.98-0.99). If comparison between pomBseen and other automated pipelines were to show comparable performance, it would confirm that pomBseen works robustly, which has already been demonstrated. If it were to show a discrepancy between pomBseen and other automated pipelines, it would demonstrate that the other pipelines fail to robustly recapitulate manual annotation, which, although perhaps of interest to users of those pipelines, is not relevant to our work.

Reviewer 2: The author(s) of article taken all my comments into careful consideration and have made improvements to the manuscript accordingly. The revised article improved the overall quality of the manuscript.

We appreciate the reviewers acknowledgement of our response to their constructive comments and suggestions.

Reviewer 3: It is my opinion that the work as it currently stands is suitable for publication. While I agree with the other reviewer that benchmarking would be a worthwhile addition, I also believe that the authors have provided enough for future researchers to perform that work if they seek to compare the efficacy of different tools/methods.

We appreciate the reviewers acknowledgement of our response to their constructive comments and suggestions and emphasize their agreement with our contention that the Review 1's request for benchmarking, although a reasonable suggestion that someone might want to do, is beyond what is required for publication in PLoS ONE.

Editor: Thank you to the reviewer for highlighting a critical concern regarding the benchmarking and evaluation of the proposed method in the manuscript titled "pomBseen: An automated pipeline for analysis of Fission Yeast images." After carefully considering the reviewer's comment, I agree that the manuscript lacks a crucial aspect of performance evaluation, specifically the comparison of the proposed method with other automated tools commonly used in the field.

To ensure that readers can properly assess the performance of the model, it is necessary to include a benchmarking analysis against existing automated methods. Additionally, the comparison should extend beyond just length measurements and should also evaluate the instance segmentation output of the model.

Reviewer 1's concern is that we did not benchmark our method against other automated image analysis packages fundamental misunderstanding of the purpose of our work. Our goal in creating and publishing pomBseen is to provide a tool for the analysis of image data that is as good as, but more efficient than, the current standard of analysis, which is manual measurement using ImageJ. I concede that, in the field of computational image analysis, benchmarking against other packages maybe the standard. However, my lab does not work in that field. We work in the field of yeast cell biology, and the standard in our field is manual image analysis, as demonstrated by the fact that we have used that approach in all of our recent work (PMIDs 33683349, 30957637, 28479325). Therefore, pomBseen is a robust and useful tool for work in our field, and we have adequately demonstrated as much in our manuscript.

In particular, I contest the Editors claim that “[t]o ensure that readers can properly assess the performance of the model, it is necessary to include a benchmarking analysis against existing automated methods”. Such benchmarking is not required to assess the performance of pomBseen. What is required in the benchmarking against the ground truth of manual annotation, which we provide.
