# Supplementary material for: pomBseen: An automated pipeline for analysis of fission yeast images
Source: PLoS One. 2023 Sep 12;18(9):e0291391. doi: 10.1371/journal.pone.0291391 (PMC10497161; doi:10.1371/journal.pone.0291391)
Supplement: Attachment — Submitted filename: revision_KSY_080723.docx [file pone.0291391.s006.docx]

Briefly, the manuscript described development of an automated analysis pipeline, to measure the length and fluorescence intensity of yeast cells and their nuclei. Using programming language MATLAB for image-analysis which is widely spread and is interesting approach, however, there are some issues to be addressed in this current manuscript. My comments are below.

1. The authors should edit figures and results more comprehensive and precise. Figures seems to be insufficient of explanation of the abbreviation without consideration for readers.

2. The authors should offer a more comprehensive explanation of the improvement points compared to the references presented in the Introduction section. For instance, Baybay EK et al. described the 3D segmentation of radially symmetric cells, which involved the combination of 2D brightfield segmentation with 3D morphological extrapolation using fission yeast (Reference #4). However, the authors did not provide the limitations of this research and a detailed explanation of how their pipeline improves upon the existing approach.

3. In the Method section, the authors descripted about the images analyzed for study. A couple of strains of cells from reference (#17), however, the strains of the reference were for functional examination of the Cdc 13 and Cdc 25 expression. The authors should explain about the reason for choosing the specific strains for their study.

4. Furthermore, the authors should provide the number of image data used for validating PomBseen and more detailed calculating methods, catering to future users rather than programmers.

5. The authors should thoroughly describe the significance of employing the MATLAB program for image analysis in comparison to other methods. This aspect constitutes a key strength of the manuscript.
